# No- and Low-Flow Time During Periprocedural Complication in Transcatheter Aortic Valve Replacement and Surgical Aortic Valve Replacement

**DOI:** 10.21470/1678-9741-2020-0473

**Published:** 2022

**Authors:** Ignazio Condello, Giuseppe Santarpino, Giuseppe Speziale

**Affiliations:** 1 Department of Cardiac Surgery, Anthea Hospital, GVM Care & Research, Bari, Italy. E-mail: ignicondello@hotmail.it; 2 Paracelsus Medical University, Nuremberg, Germany; 3 Department of Experimental and Clinical Medicine, Cardiac Surgery Unit, “Magna Graecia” University of Catanzaro, Italy

The duration of resuscitation may be defined as the sum of two distinct intervals: 1) no-flow ([NF], interval from collapse to initiation of CPR); and 2) low-flow ([LF], interval from start of cardiopulmonary resuscitation [CPR] to return of spontaneous circulation [ROSC] or termination of resuscitation). Relatively few published studies have examined the impact of low-flow and no-flow intervals on clinical outcomes^[1]^. Transcatheter aortic valve replacement (TAVR) is a complex procedure and often associated with complications that may result in hemodynamic collapse. The cardiocirculatory supports are essential to manage complications during interventional cardiology and cardiac surgery procedures, in particular to reduce the no-flow and low-flow times. Despite using extracorporeal supports, crucial differences coexist between TAVR and surgical aortic valve replacement (SAVR). In our experience, the no-flow time without hemodynamic support has a low incidence in periprocedural complication during surgical aortic valve replacement, especially from anatomical and structural alterations. This is favored by the presence, in surgical practice, of cardiopulmonary bypass, surgical access to cardiac structures, mechanical ventilation and access for monitoring and infusion of drugs. In this context, we read with great interest the article “Emergency use of cardiopulmonary bypass in complicated transcatheter aortic valve replacement: importance of a heart team approach” by Roselli et al.^[2]^, which described the indications for the use of salvage cardiopulmonary bypass (CPB) and assess the outcomes in life-threatening complications during TAVR in high-risk patients. Three hundred and three patients underwent TAVR, and 12 (4%) required emergency CPB. The main indication for CPB was hemodynamic instability with or without ischemic changes. These resulted from aortic insufficiency (n=5), valve embolization (n=3), coronary malperfusion (n=2), bleeding requiring pericardiocentesis (n=1), and bleeding from ventricular apex (n=1).

The Randomized Controlled Trial Partner III concluded that, among patients with severe aortic stenosis and low surgical risk, the rate of the composite endpoint of death, stroke, or rehospitalization at 1 year was significantly lower with TAVR than with surgery[^3^]. In this trial, there were six deaths during the index hospitalization, which occurred in two patients in the TAVR group and in four patients in the surgery group. Other serious intraprocedural complications that occurred in the TAVR group included implantation of a second valve, annulus rupture, coronary-artery obstruction, and ventricular perforation^[3]^. From our point of view, it may be interesting to investigate, in these circumstances, the survival between TAVR and SAVR in periprocedural complications in terms of management superiorities during no- or low-flow times and to analyze the results and outcomes after resuscitation.
